# An Ultrahigh Frequency Partial Discharge Signal De-Noising Method Based on a Generalized S-Transform and Module Time-Frequency Matrix

**DOI:** 10.3390/s16060941

**Published:** 2016-06-22

**Authors:** Yushun Liu, Wenjun Zhou, Pengfei Li, Shuai Yang, Yan Tian

**Affiliations:** 1 School of Electrical Engineering, Wuhan University, No. 299, Bayi Road, Wuhan 430072, China; silencelys@126.com (Y.L.); pengfei9966@126.com (P.L.); ys3254@163.com (S.Y.); 2 Guangzhou Power Supply Bureau Co. Ltd., No. 38, Huangshidong Road, Guangzhou 510620, China; babyyan22@163.com

**Keywords:** ultrahigh frequency antenna sensor, partial discharge, signal de-noising, generalized S-transform, module time-frequency matrix, periodic narrowband noise, Gaussian white noise

## Abstract

Due to electromagnetic interference in power substations, the partial discharge (PD) signals detected by ultrahigh frequency (UHF) antenna sensors often contain various background noises, which may hamper high voltage apparatus fault diagnosis and localization. This paper proposes a novel de-noising method based on the generalized S-transform and module time-frequency matrix to suppress noise in UHF PD signals. The sub-matrix maximum module value method is employed to calculate the frequencies and amplitudes of periodic narrowband noise, and suppress noise through the reverse phase cancellation technique. In addition, a singular value decomposition de-noising method is employed to suppress Gaussian white noise in UHF PD signals. Effective singular values are selected by employing the fuzzy c-means clustering method to recover the PD signals. De-noising results of simulated and field detected UHF PD signals prove the feasibility of the proposed method. Compared with four conventional de-noising methods, the results show that the proposed method can suppress background noise in the UHF PD signal effectively, with higher signal-to-noise ratio and less waveform distortion.

## 1. Introduction

Partial discharge (PD) is a major cause and manifestation of insulation degradation. PD detection has been utilized for insulation condition assessment of high voltage (HV) apparatus [1]. When PD occurs in a HV apparatus, ultra-high frequency (UHF) electromagnetic waves are radiated from the PD source and propagate to the space of the substation through objects with no shielding effect [2]. External UHF antenna sensors can be utilized to detect UHF PD signals [3], however, in field PD tests, the UHF PD signals detected by antenna sensors are vulnerable to interference due to the numerous electromagnetic waves present in space [4]. The background noise interferes with the PD detection and causes PD pulse shapes to be distorted, which may negatively affect HV apparatus fault diagnosis and localization accuracy [5,6].Therefore, noise suppression is a vital issue of field UHF PD signal testing.

The background noise mainly includes the periodic narrowband noise and white Gaussian noise [7]. The periodic narrowband noise generally originates from wireless communication systems and may overwhelm the original PD signals. Several techniques have been proposed to suppress the narrowband noise [8,9,10,11,12,13]. Methods based on adaptive filters have been proposed to effectively suppress the narrowband noise [9,10], however, when the frequencies of the interferences and the PD signal are mixed, the de-noised PD signal is distorted due to the calculation error of the Fourier transform spectral analysis and the characteristics of the impulse response filter [11]. The methods based on wavelet transform (WT) were reported to extract PD signals from excessive narrowband noise [12], but due to the diversity of detected UHF PD signals, it is difficult to select the suitable mother wavelet and the number of decomposition-reconstruction levels [13].

The Gaussian white noise is generated by the heating effect of HV apparatus and will distort the UHF PD signals. Many methods have been proposed to suppress the white noise, such as the WT method [14], empirical mode decomposition method [15], and mathematical morphology method [16]. Recently, a novel method based on singular value decomposition (SVD) has been utilized for Gaussian white noise suppression, and more accurate recovery of the original PD signal was obtained [17]. A hard thresholding method based on standard deviation was employed to select effective singular values in this method.

In this paper, a novel de-noising method based on the module time-frequency matrix (MTFM) is proposed to suppress periodic narrowband noise and Gaussian white noise. The MTFM of detected UHF PD signals is constructed by employing the generalized S-transform time-frequency analysis technique. Frequencies and amplitudes of periodic narrowband noise can be calculated by utilizing the sub-matrix maximum module value method. The periodic narrowband noise is separated from the noisy UHF PD signals through the reverse phase superposition of the MTFM. Different from the conventional SVD de-noising method in [17,18], the singular values is calculated through decomposing the 2-dimensional MTFM. The fuzzy c-means (FCM) clustering method is employed to select effective singular values for recovering the UHF PD signals without Gaussian white noise. The de-noising results of simulated and field test UHF PD signals demonstrate the validity of the proposed method.

## 2. Algorithm of the Generalized S-Transform

As a non-stationary signal, the localized feature information of an UHF PD signal cannot be expressed only in time domain or frequency domain [19,20]. To extract information related to the time-frequency variation of a PD signal, Stockwell proposed the S-transform [21] to map time domain signal into the time-frequency domain, the S-transform of signal *x*(*t*) is defined as:

(1) $$S(\tau ,f)=\int_{-\infty }^{\infty }x(t)w(t-\tau ,f)e^{-j2\pi ft}dt$$

(2) $$w(t-\tau ,f)=\frac{\left|f\right|}{\sqrt{2\pi }}e^{-\frac{f^{2}{\left(t-\tau \right)}^{2}}{2}}$$

where *t* and *τ* are time, *f* is frequency, *w*(*t − τ*, *f*) is the Gaussian window function. The inverse S-transform is expressed as:

(3) $$x(t)=\int_{-\infty }^{\infty }\left[\int_{-\infty }^{\infty }S(\tau ,f)\right]e^{j2\pi ft}df$$

The S-transform combines the separate strengths of the short-time Fourier transform and WT, which has provided an alternative approach to process non-stationary signals [22]. Because the height and the width of Gaussian window are varied by changing the frequency, the S-transform overcomes the defect of constant time-frequency resolution of the short time Fourier transform [23].

Because the form of the S-transform window function is invariant, the application is limited in some cases. Pinnegar proposed a generalized S-transform [24] by adding an adjustable factor *λ* to the Gaussian window function as the Equation (4) shown:

(4) $$w(t-\tau ,f,\lambda )=\frac{\left|\lambda f\right|}{\sqrt{2\pi }}e^{-\frac{\lambda^{2}f^{2}{\left(t-\tau \right)}^{2}}{2}}$$

where *λ* is the adjustable factor and *λ* > 0. Then the generalized S-transform is obtained as:

(5) $$G(\tau ,f,\lambda )=\int_{-\infty }^{\infty }x(t)w(t-\tau ,f,\lambda )e^{-j2\pi ft}dt$$

In the generalized S-transform, *λ* < 1 corresponds to high frequency resolution, *λ* > 1 corresponds to high time resolution, *λ* = 1 corresponds to the standard S-transform window. In the application of generalized S-transform, an appropriate value of *λ* to the actual situation can be chosen to modify the time-frequency resolution.

According to Equation (5), as *f*→*n*/*NT*, *τ*→*iT*, the discrete form of the generalized S-transform is expressed as:

(6) $$\left\{ \begin{array}{l} G(iT,\frac{n}{NT},\lambda )=\sum_{m=0}^{N-1}X\left[\frac{m+n}{NT}\right]\cdot e^{-2\pi^{2}m^{2}/\lambda^{2}n^{2}}e^{j2\pi mi/N},n\neq 0\\ G(iT,0)=\frac{1}{N}\sum_{m=0}^{N-1}x(\frac{m}{NT}),n=0 \end{array}\right.$$

where *T* is the sampling interval, *N* is the total number of sample points.

The generalized S-transform of *x*(*t*) is a 2-dimensional complex matrix in time-frequency domain, where the columns correspond to time sampling points and the rows correspond to time frequency sampling points [25]. For simplifying the calculations, the MTFM can be obtained by modeling every element in the complex matrix. The time-frequency distribution based on the MTFM reflects the time-frequency features of UHF PD signal. These features contribute to the analysis of the periodic narrowband noise [26].

## 3. De-Noising Method of Periodic Narrowband Noise

### 3.1. Simulated Signals

**Figure 1:**
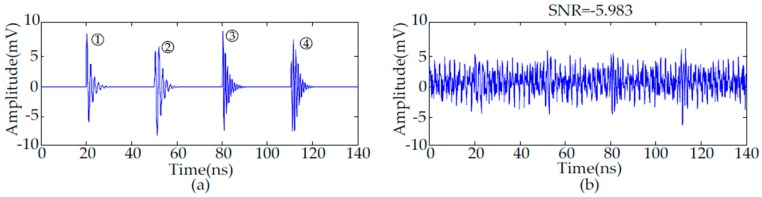
Simulated UHF PD signals: (**a**) Simulated PD signals; (**b**) Simulated noisy PD signals.

Due to the noise interference in field-detected UHF PD signals, the original PD signal cannot be obtained for illustrating the procedure and verifying the feasibility of the proposed method. An UHF PD signal is a type of transient signal with a short time duration [11]. Therefore, the single exponential decay oscillating impulses (pulses 1 and 2) and double exponential decay oscillating impulses (pulses 3 and 4) are utilized to simulate four types of UHF PD signals [12,13]. Equations (7) and (8) give the corresponding mathematical models, respectively:

(7) $$Z_{1}(t)=A_{1}e^{\frac{-t}{\tau }}\sin2\pi f_{c}t$$

(8) $$Z_{2}(t)=A_{2}(e^{\frac{-1.3t}{\tau }}-e^{\frac{-2.2t}{\tau }})\sin2\pi f_{c}t$$

where *A* is the amplitude, *τ* is the decay coefficient and *f*_c_ is the oscillation frequency. Table 1 gives the parameters of each PD pulse. The simulated sampling frequency of each pulse is 10 Gs/s. Figure 1a shows the four types of UHF PD signals.

The waveform of practical periodic narrowband noise is sinusoidal with constant amplitude [27]. Equation (9) gives the mathematical model of periodic narrowband noise, where *A* is the amplitude assumed to be 2 mV. Based on the frequencies of wireless communication signals in China, *f_i_* is the frequency assumed to be 470 MHz, 900 MHz, 1800 MHz, respectively. The simulated UHF PD signals with periodic narrowband noise and Gaussian white noise are given in Figure 1b. The SNR of these noisy PD signals is −5.983 dB:

(9) $$Z_{3}(t)=A\sum_{i=1}^{n}\sin(2\pi f_{i}t)$$

### 3.2. Calculation Method of Frequency and Amplitude

#### 3.2.1. Sub-Matrix Maximum Module Value Method

**Figure 2:**
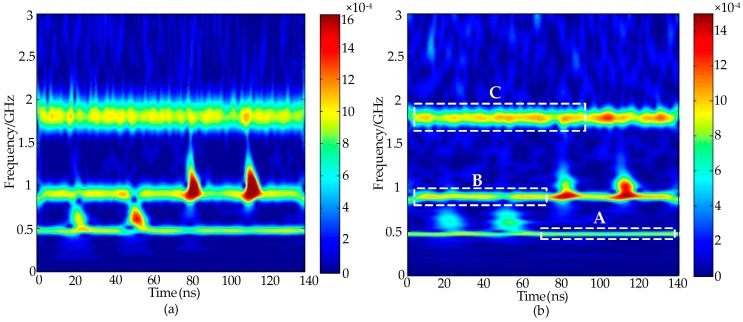
The time-frequency distribution of the noisy PD signals calculated by (**a**) the S-transform; and (**b**) the generalized S-transform.

When the frequencies of the periodic narrowband noise and PD signals are partially overlapped, the PD pulse(s) are mixed with strong narrowband noise in a time-frequency distribution calculated by the S-transform, meaning that it is difficult to separate the narrowband noise from the PD pulse(s) [26]. The MTFM of the noisy PD signals calculated by the S-transform and the generalized S-transform (*λ* = 0.3), respectively, are drawn as a time-frequency distribution contour map in Figure 2. The comparison results show that higher frequency resolution can be obtained by utilizing the generalized S-transform (*λ* < 1), which may contribute to extract the narrowband noise features [26].

The time-frequency distribution of periodic narrowband noise is concentrated, with long duration, and the frequency distribution of a PD signal is discrete, with short duration. Therefore, both types of signals can be recognized in the time-frequency distribution. Due to the influence of Gaussian white noise, the accurate frequencies and amplitudes of periodic narrowband noise cannot be obtained directly from the time-frequency distribution. In this paper, a novel sub-matrix maximum module value (SMMV) method is proposed for calculating the frequencies and amplitudes of narrowband noise. The SMMV method is described as follows:

Step 1: As shown in Figure 2b, parts of the periodic narrowband noise are divided into Regions A, B, C, and the corresponding localized time-frequency sub-matrices are extracted;
Step 2: Search the coordinate positions of the maximum module value points in each column of each sub-matrix;
Step 3: Based on the MTFM calculated by the generalized S-transform (λ < 1), record the corresponding frequency values of the maximum module value points (*f*_1_, *f*_2_, *f*_3_, …, *f*_n_) and the calculated time of each frequency value (*t*_1_, *t*_2_, *t*_3_, …, *t*_n_);
Step 4: Calculate the frequencies *f*_p_ of each periodic narrowband noise based on Equation (10):
  (10) $$f_{p}=\frac{\sum_{k=1}^{n}f_{k}t_{k}}{n}$$
Step 5: Record the module value of each sampling point (*m*_1_, *m*_2_, …, *m*_n_) in time on the calculated frequencies in Step (4), and calculate the module value *m*_p_ of each corresponding narrowband noise in the MTFM based on Equation (11):
  (11) $$m_{p}=\frac{\sum_{k=1}^{n}m_{k}}{n}$$
Step 6: Calculate the amplitudes of periodic narrowband noise by utilizing the inverse generalized S-transform, and reconstruct time domain waveform based on Equation (3).

#### 3.2.2. Selection of the Adjustable Factor *λ*

In general, the generalized S-transform is a modified Fourier transform method, and the time-frequency resolution is still restricted by the Heisenberg uncertainty principle. This means that the time resolution decreases when the frequency resolution increases in a time-frequency distribution. Therefore, an appropriate value of *λ* contributes to the calculation of more accurate frequencies and amplitudes of periodic narrowband noise based on the SMMV method. Different values of *λ* were used to calculate the amplitudes and frequencies of each narrowband noise, with the results given in Table 2. 

**Figure 3:**
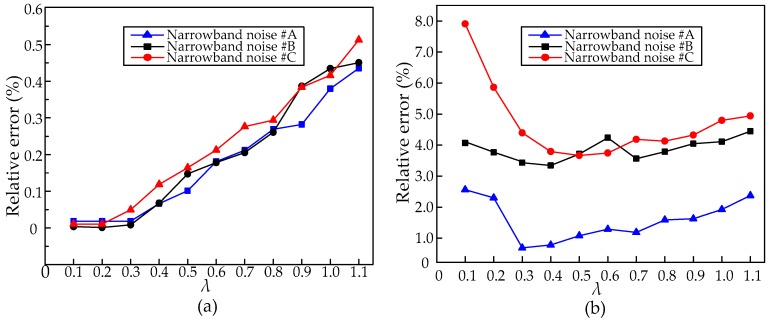
Relative errors by using different value of λ: (**a**) frequency; (**b**) amplitude.

Compared with the original assumed value in the simulation, the relative errors of different calculated narrowband noise are given in Figure 3. Due to the improvement of frequency resolution, the relative errors of the calculated frequencies are reduced with the decrease in the value of *λ*. However, due to the reduction of time resolution, the relative errors of the calculated amplitudes are increased when the value of *λ* is lower than 0.3. The relative error is defined as:

(12) $$\mathrm{Relative}\mathrm{error}=\frac{\left|O-C\right|}{O}\times 100\%$$

where *O* and *C* are the amplitude (frequency) of the original and calculated periodical noise, respectively. The results show that the value of *λ* can be set between 0.3 and 0.4 to accurately calculate the frequencies and amplitudes of periodic narrowband noise. The value of λ is set to 0.3 in this paper.

### 3.3. Method of Periodic Narrowband Noise Suppression

To suppress the periodic narrowband noise, the MTFM based on the generalized S-transform is applied as follows:

Step 1: Use the generalized S-transform to obtain the MTFM ***S****_M_*_×*N*_ and time-frequency distribution of noisy UHF PD signals, where ***S****_M×N_* is the matrix with *M* rows and *N* columns ;
Step 2: Extract the localized matrices of periodic narrowband noise without PD signals based on the time-frequency distribution;
Step 3: Obtain the amplitudes and frequencies of periodic narrowband noise utilizing the above-described SMMV method;
Step 4: Use the generalized S-transform to calculate the MTFM ***P***_1_, ***P***_2_, …, ***P***_n_ of periodic narrowband noise based on Equation (6) and the results of Step (3), where ***P****_k_* is the matrix with *M* rows and *N* columns;
Step 5: Suppress the periodic narrowband noise by utilizing the reverse phase cancellation method is given in Equation (13), where ***W****_M_*_×*N*_ is the MTFM without periodic narrowband noise:
  (13) $$W_{M\times N}=S_{M\times N}-\sum_{k=1}^{n}P_{k}$$
Step 6: Use the inverse generalized S-transform to obtain the de-noised UHF PD signals in time domain of the ***W****_M×N_*.

**Figure 4:**
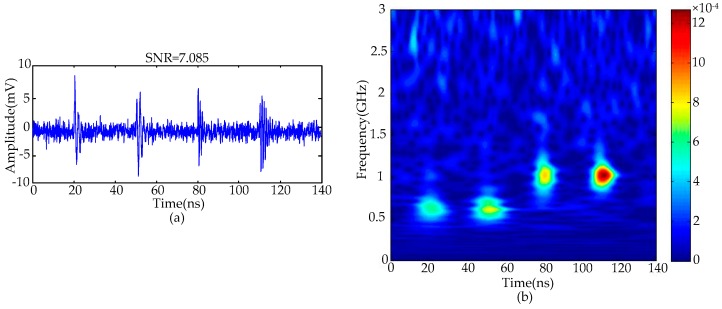
UHF PD signals with suppressed periodic narrowband noise: (**a**) in time domain; (**b**) in time-frequency domain.

Figure 4 gives the de-noising results of simulated noisy UHF PD signals utilizing the proposed method. The de-noising results indicate that periodic narrowband noise has been suppressed effectively and only Gaussian white noise exists in the UHF PD signals. The SNR of this signal is 7.085 dB.

## 4. De-Noising Method of Gaussian White Noise

### 4.1. Singular Value Decomposition De-Noising Method

**Figure 5:**
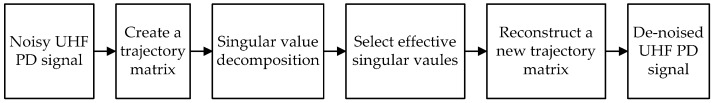
Procedure of the conventional SVD de-noising method.

Suppression of Gaussian white noise is vital to extract accurate UHF PD pulse waveforms. The singular value decomposition (SVD) de-noising method is a nonlinear filtering method, which was shown to be capable of effectively suppressing white noise [17,28]. The conventional SVD de-noising method procedure is given in Figure 5 and is illustrated as follows.

Step 1: For a signal sequence *x*(*i*), *i* = 1, 2, …, *N*, a trajectory matrix of *x*(*i*) is defined as
  (14) $$A=\left[\begin{array}{cccc} x(1) & x(2) & \cdots & x(n)\\ x(2) & x(3) & \cdots & x(n+1)\\ \vdots & \vdots & \vdots & \vdots \\ x(N-n+1) & x(N-n+2) & \cdots & x(N) \end{array}\right]$$
  where ***A***∈***R****^m×n^*, 1 < *n* < *N*, and *m* = *N* – *n* + 1.
Step 2: The SVD of this real matrix ***A***∈***R****^m^*^×*n*^ is defined as:
  (15) $$A=U\Lambda V^{T}$$
  where ***U*** and ***V*** are the orthogonal matrix, ***U***∈***R****^m^*^×*n*^, ***V***∈***R****^m^*^×*n*^. ***Λ*** = [diag(*a*_1_, *a*_2_, …, *a_q_*), ***O***] or its transposition matrix, which is determined by *m* < *n* or *m* ≥ *n*. ***O*** is the zero matrix, *q* = min(*m*, *n*), *a*_1_ ≥ *a*_2_ ≥…≥ *a_q_*. *a_i_*, *i* = 1, 2, …, *q*, are the singular values of matrix.
Step 3: In matrix ***Λ***, the effective singular values corresponding to a PD signal are reserved while the corresponding white noise are set to zero, and thus a new matrix ***Λ_re_*** can be obtained.
Step 4: A new trajectory matrix ***A***_re_ is reconstructed by utilizing ***Λ_re_***, the original ***U*** and ***V*** through Equation (16), and the de-noised PD signals are recovered from the trajectory matrix ***A_re_***:
  (16) $$A_{re}=U\Lambda_{re}V^{T}$$

### 4.2. Selection Method of Effective Singular Values

Because the trajectory matrix is created by truncating the signal in the time domain, SVD results only contain time-amplitude information. It means that the recovered de-noised PD signal may be partially distorted [18]. The MTFM of the noisy PD signals is set as the trajectory matrix ***A*** in this paper. Eigenvectors of frequency and time are represented by the orthogonal matrices ***U*** and ***V***, respectively. SVD results of the MTFM contain time-frequency-amplitude information, meaning that some characteristic quantities of another dimension can be obtained in the ***U***, ***V*** and singular values. Therefore, the recovered PD signal after de-noising will be more accurate compared with the conventional algorithm ones.

For the SVD de-noising method, the most important step is how to select the number of effective singular values, which largely determines the de-noising effect. The recovered PD signal is distorted when too small a number of effective singular values is selected, and residual white noise would remain when selecting over many effective singular values [17]. The methods proposed in [17,18] selected effective singular values by the hard thresholding technique, which may deteriorate the original UHF PD signal. In the MTFM, as Figure 4b showed, the module values corresponding to PD signal are greater with a concentrated distribution. On the contrary, the module values corresponding to Gaussian white noise are lower with a scattered distribution. Therefore, the magnitudes of singular values corresponding to PD signals are greater with a large difference. In this paper, a novel method for selecting effective singular values based on the fuzzy c-means (FCM) clustering algorithm is proposed. Features ***fe***_1_ and ***fe***_2_ are extracted as the features for clustering when selecting effective singular values. ***fe***_1_ and ***fe***_2_ are shown in Equation (17):

(17) $$\left\{ \begin{array}{l} fe_{1}=a_{1},a_{2},...,a_{n}\\ fe_{2}=b_{1},b_{2},...,b_{n},b_{i}=a_{i}-a_{i-1},i=1,2,...,q-1 \end{array}\right.$$

The basic principle of FCM can be described briefly as follows. Given a dataset of PD features ***F*** = {***fe***_1_, ***fe***_2_, ···, ***fe****_N_*}, where ***fe****_j_* = (*fe*_1_, *fe*_2_, ···, *fe_m_*)^T^. FCM aims to minimize the c-means function, which is defined as:

(18) $$\min f(UU,VV)={\sum_{i=1}^{c}\sum_{j=1}^{N}{\left(\mu_{ij}\right)}^{m}\left\|fe_{j}-v_{i}\right\|}^{2}$$

(19) $$s.t.\{\begin{array}{c} \sum_{i=1}^{c}\mu_{ij}=1,(1\leq j\leq N)\\ 0\leq \mu_{ij}\leq 1,(1\leq i\leq c,1\leq j\leq N)\\ 0\leq \sum_{j=1}^{N}\mu_{ik}\leq N,(1\leq i\leq c) \end{array}$$

where *c* is the pre-defined cluster number, ***VV*** indicates the cluster center matrix, ***v****_i_* is the cluster center of class *i*, 1 ≤ *i ≤ c*, ***UU*** = [*μ_ij_*]*_c_*_×*N*_ represents the fuzzy membership degree matrix, *μ_ij_* is the fuzzy membership degree of ***f****_j_* belong to class *i*, *m* is the weighting coefficient, and *m* is set as 2 in this paper, *d_ij_* = //***f****_j_*-***v****_i_*//^2^ represents the Euclidean distance between sample ***f****_j_* and ***v****_i_*. In order to obtain the minimum of *f* (***UU***, ***VV***) and find the optimal ***UU*** = [*μ_ij_*]*_c_*_×*N*_ and ***v****_i_*, a Lagrange multiplier is formed to solve the optimization problem:

(20) $$F=\sum_{i=1}^{c}{\left(\mu_{ij}\right)}^{m}{\left(d_{ij}\right)}^{2}-\lambda (\sum_{i=1}^{c}\mu_{ij}-1)$$

The optimal conditions of Equation (20) are:

(21) $$\{\begin{array}{c} \frac{\partial F}{\partial \lambda }=\sum_{i=1}^{c}\mu_{ij}-1=0\\ \frac{\partial F}{\partial \mu_{ij}}=[m{\left(\mu_{ij}\right)}^{m-1}{\left(d_{ij}\right)}^{2}-\lambda ]=0 \end{array}$$

According to Equation (21), *μ_ij_* is calculated uing Equation (22). Then by solving $\frac{\partial f(UU,VV)}{\partial v_{i}}=0$, ***v****_i_* is calculated as in Equation (23). More details of the FCM method can be found in [29]:

(22) $$\mu_{ij}={\sum_{k=1}^{c}\left(\frac{d_{ij}}{d_{kj}}\right)}^{\frac{2}{1-m}}$$

(23) $$v_{i}=\frac{\sum_{j=1}^{N}{\left(\mu_{ij}\right)}^{m}\cdot fe_{j}}{\sum_{j=1}^{N}{\left(\mu_{ij}\right)}^{m}}$$

### 4.3. De-Noising Method of Gaussian White Noise

The suppression procedure of Gaussian white noise based on the MTFM and FCM clustering involves the following steps:

Step 1: Calculate singular values of the MTFM of the noisy PD signals by SVD;
Step 2: Calculate features of singular values sequence based on Equation (17);
Step 3: Classify singular values into two groups utilizing FCM clustering algorithm, which represent UHF PD signals and Gaussian white noise, respectively;
Step 4: Select the group whose maximum value is greater as the effective singular values, the number of effective singular values is defined as *k* in this paper;
Step 5: Recover the de-noised PD signal by the conventional SVD de-noising method.

**Figure 6:**
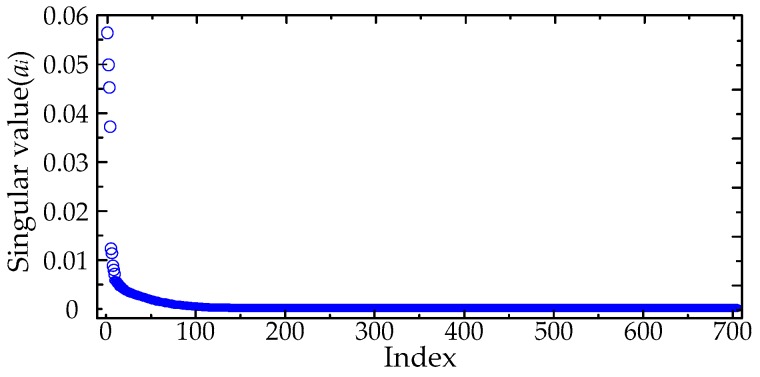
Calculated singular values by decomposing the MTFM.

**Figure 7:**
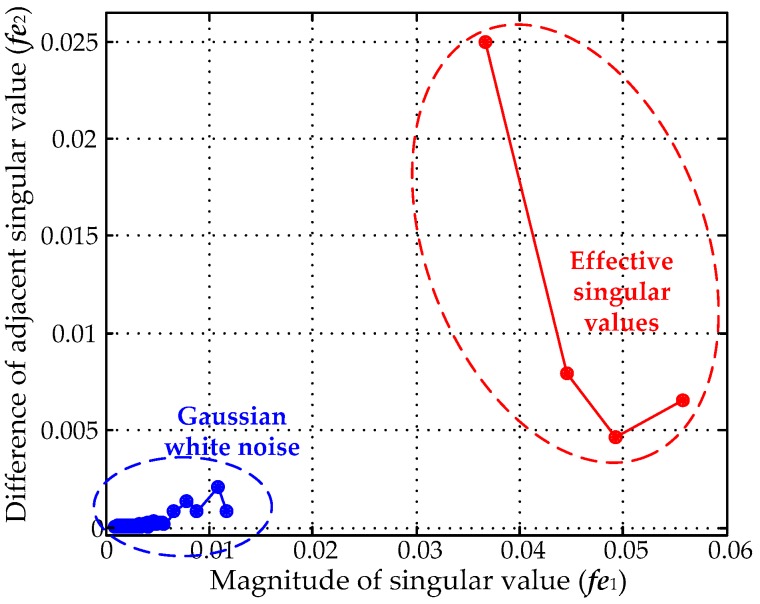
Selection results of effective singular values based on the FCM clustering algorithm.

**Figure 8:**
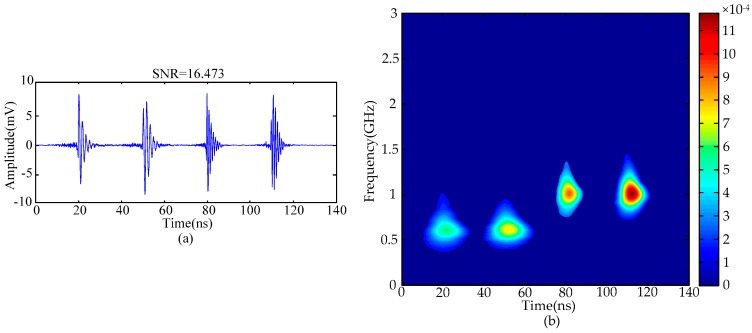
De-noised PD signals: (**a**) time-domain signals; (**b**) time-frequency distribution.

**Figure 9:**
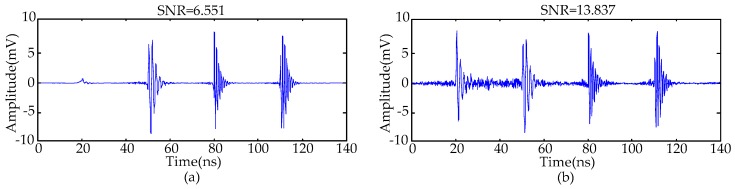
De-noised PD signals employing SVD when (**a**) *k* = 3; and (**b**) *k* = 5.

The calculated singular values of the MTFM are given in Figure 6, and the classification result utilizing the FCM clustering algorithm is given in Figure 7. The value of *k* was set as 4 in this paper. The de-noising results are given in Figure 8. The results show that Gaussian white noise is suppressed successfully by using the proposed method. When the number of effective singular values was set to *k* − 1 and *k* + 1, the de-noised PD signals are given in the Figure 9. The first pulse of recovered PD signals is distorted severely when *k* = 3, and the SNR of the de-noised signals will be lowered when *k* = 5. The comparison of results indicates that the proposed method based on FCM clustering algorithm is capable of selecting effective singular values. 

## 5. Simulation De-Noising Results and Discussions

**Figure 10:**
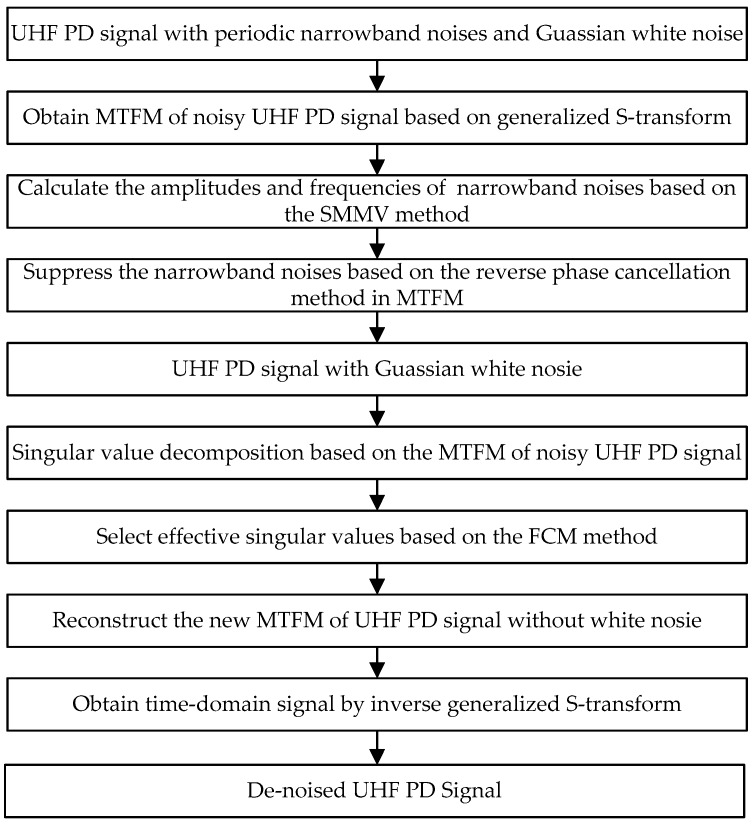
Procedure of the proposed de-noising method.

**Figure 11:**
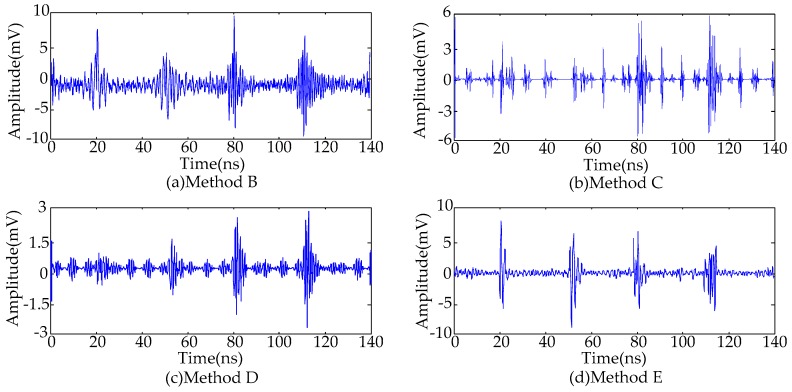
De-noising results by employing each method: (**a**) Method B; (**b**) Method C; (**c**) Method D; and (**d**) Method E.

The procedure of the proposed de-noising method is summarized in Figure 10. For comparison, four conventional de-noising methods [9,10,11,12,13,17] were employed to suppress the noisy UHF PD signals shown in Figure 1b. The types of noise and de-noising techniques are shown in Table 3. The db2 and db8 wavelets are selected as the mother wavelet in Methods C and D where the decomposition level was set as 10. The de-noised PD signals are given in Figure 11. To compare the performance of various de-noising methods, the following evaluation parameters are introduced [14,30,31].

(I) The SNR illustrates the effectiveness of noise suppression, which is defined as:

(24) $$\text{SNR}=10{\text{log}}_{10}(\frac{\sum_{i=1}^{N}{\left|s(i)\right|}^{2}}{\sum_{i=1}^{N}{\left|r(i)-s(i)\right|}^{2}})$$

where *s*(*i*) is the original signal and *r*(*i*) is the de-noised signal. Higher SNR value means more effective background noise suppression.

(II) The root-mean-square error (RMSE) is defined as:

(25) $$\text{RMSE}=\sqrt{\frac{1}{N}\sum_{i=1}^{N}{\left|r(i)-s(i)\right|}^{2}}$$

RMSE is used to evaluate waveform distortion of de-noised signal compared with the original signal. Lower RMSE value means less waveform distortion of UHF PD signal waveform.

(III) The normalized correlation coefficient (NCC) is defined as:

(26) $$\text{NCC}=\frac{\sum_{i=1}^{N}s(i)\cdot r(i)}{\sqrt{(\sum_{i=1}^{N}s^{2}(i))\cdot (\sum_{i=1}^{N}r^{2}(i))}}$$

NCC is used to evaluate waveforms similarity between the original and de-noised signal. The NCC value is between the negative one (−1) and positive one (+1), −1 means fore-and-aft waveform reverse, zero means orthogonal, and +1 means almost the same.

(IV) The variation trend parameter (VTP) is composed of rise variation trend parameter (RVTP) and fall variation trend parameter (FVTP).

(27) $$\text{RVTP}=\frac{\sum_{i=2}^{N}\left[r(i)-r(i-1)\right]}{\sum_{i=2}^{N}\left[s(i)-s(i-1)\right]}$$

where *s*(*i*) > *s*(*i* − 1), *r*(*i*) > *r*(*i* − 1).

(28) $$\text{FVTP}=\frac{\sum_{i=2}^{N}\left[r(i-1)-r(i)\right]}{\sum_{i=2}^{N}\left[s(i-1)-s(i)\right]}$$

where *s*(*i*) < *s*(*i* − 1), *r*(*i*) < *r*(*i* − 1).

VTP is the mean value of RVTP and FVTP.

(29) $$\text{VTP}=\frac{\text{RVTP}+\text{FVTP}}{2}$$

VTP is used to describe the similarity of the wave variation tendency, which measures the oscillating situation of the waveform. When the VTP value is close to 1, the variation tendency of two waveforms is the most similar.

Calculated results of various evaluation parameters are given in Table 4. The comparison results of de-noised PD signals and evaluation parameters show that:

(1) The adaptive filtering method is capable of suppressing periodic narrowband noise. However, because the frequencies of narrowband noise and UHF PD signals are mixed, the de-noised PD signal waveforms are distorted.
(2) Due to the large variation in time-frequency characteristics of UHF PD signals, de-noising results are affected by the mother wavelet selection [11]. In this paper, the de-noised signals with lower SNR and larger waveform distortion are obtained by utilizing Method C (select db2 wavelet); the de-noised signals with higher SNR and less waveform distortion are obtained by utilizing Method D (select db8 wavelet). Therefore, the de-noising effectiveness when using wavelet decomposition may become worse when an unsuitable mother wavelet is selected.
(3) Because the effective singular values calculated from the MTFM represent more time-frequency-amplitude information, the proposed method in this paper is capable of obtaining the de-noised UHF PD signals with higher SNR and less waveform distortion compared with conventional SVD de-noising method.
(4) Compared with four conventional de-noising methods, the proposed method based on the MTFM is capable of suppressing the periodic narrowband noise and Gaussian white noise successfully. Hence, by the proposed method, the de-noised UHF PD signals are similar to the original signals.

**Figure 12:**
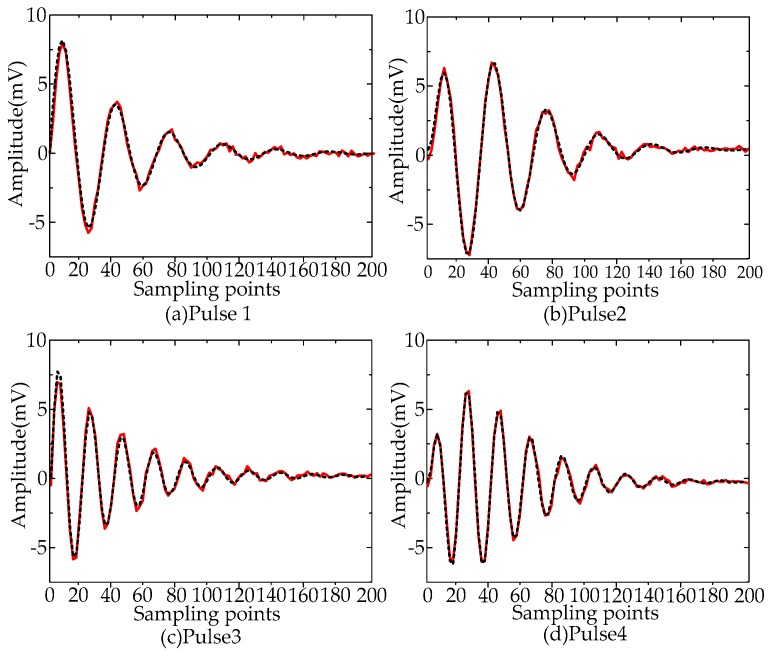
Comparisons of the original and the de-noised PD pulse shapes. (**a**) Pulse 1; (**b**) Pulse 2; (**c**) Pulse 3; (**d**) Pulse 4.

The pulse shapes of de-noised UHF PD signal utilizing Method A are given in Figure 12. The original and the de-noised PD pulses were drawn in the black dashed line and red solid line, respectively. The results show that the de-noising method proposed in this paper can recover the original PD pulses successfully and thus can assist in feature parameters extraction of UHF PD signals for insulation defect type recognition.

## 6. De-Noising Results of Field Detected Signals

**Figure 13:**
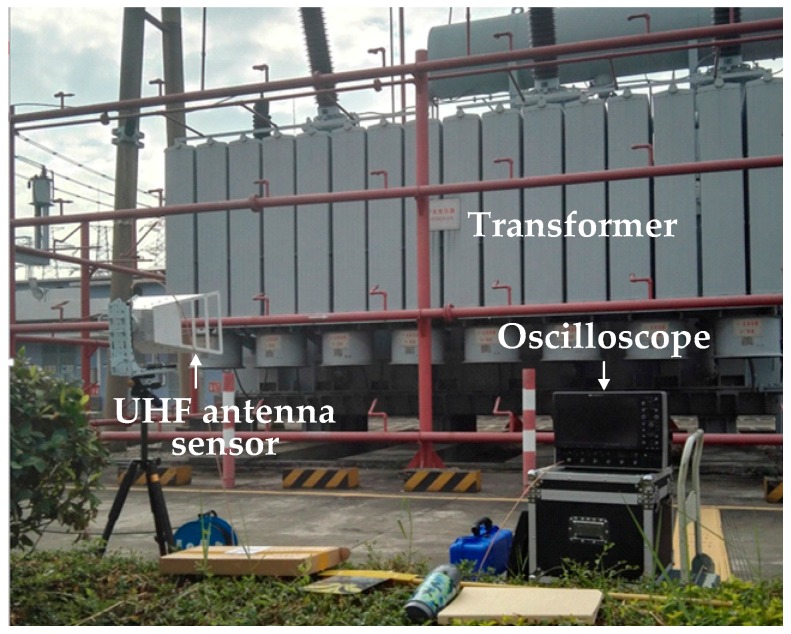
Field detection setup in substation.

**Figure 14:**
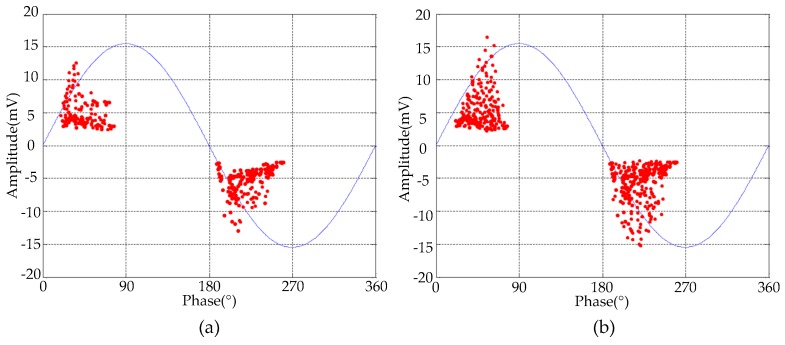
PRPD patterns corresponding to the detected UHF signals: (**a**) signal #1; (**b**) signal #2.

**Figure 15:**
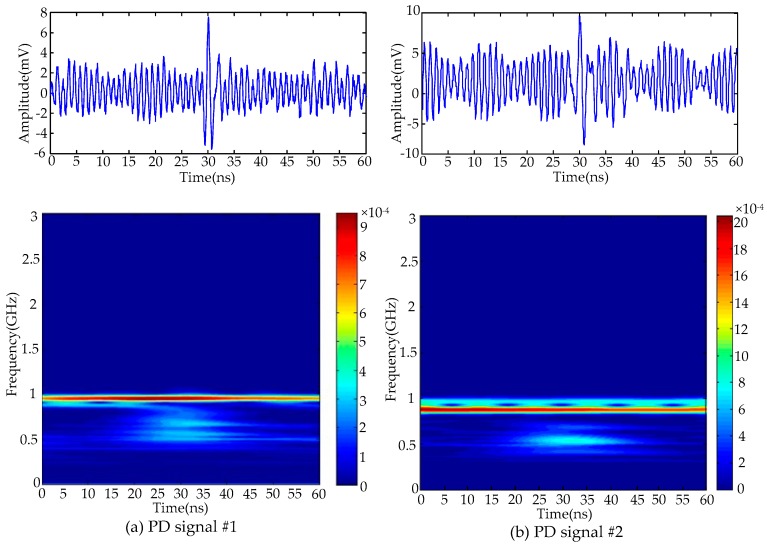
Waveforms and time-frequency distributions detected UHF PD signals: (**a**) signal #1; (**b**) signal #2.

To verify the effectiveness of the proposed de-noising method for field detected PD signals, two UHF PD signals were detected from two 500 kV substations in Guangzhou, China. The picture of the field detection setup is given in Figure 13. An external horn antenna sensor (AInfo-LB530NF, A-Info Technology Co., Ltd, Chengdu, China) was employed to detect the PD signals. The operation bandwidth of antenna sensor was 0.5–3 GHz, and the maximum antenna gain was 11 dBi. The detected UHF PD signals were recorded using a digital oscilloscope (Lecory-WavePro740, Teledyne LeCroy, New York, NY, USA), which has 4 GHz bandwidth, 20 GSamples/s sampling rate, central processing unit (CPU) with 2.6 GHz dominant frequency and random access memory (RAM) with 2 G memory. The phase resolved partial discharge (PRPD) patterns corresponding to two detected UHF signals are given in Figure 14, and according to [20,31], the detected signals were originated from the PD sources. The time-domain waveforms and time-frequency distributions of two detected PD signals are given in Figure 15. It is obviously that narrowband noise and white noise interfered with the detected PD signals severely.

The calculated frequencies and amplitude of periodic narrowband noise employing the SMMV method are given in Table 5. 

**Figure 16:**
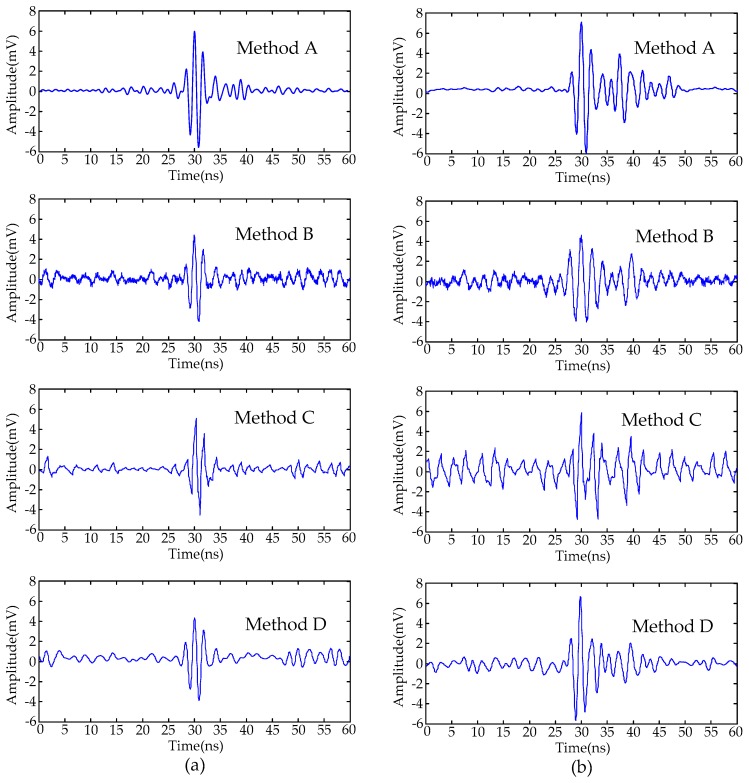
De-noised PD signals in field detection using each method: (**a**) signal #1; (**b**) signal #2.

Periodic narrowband noise originates in local mobile phone wireless communication signals. The de-noising results of field detected PD signals by the methods in Table 3 are given in Figure 16. Because the original PD signal without noise is not obtained as a reference in field detection, the evaluation parameters introduced in Equations (24)–(29) are not available. Therefore, noise reduction ratio (NRR) and the amplitude reduction ratio (ARR) are proposed to evaluate the de-noising results [32], which are defined as:

(30) $$\text{NRR}=10(\log_{10}\sigma_{1}^{2}-\log_{10}\sigma_{2}^{2})$$

(31) $$\text{ARR}=\frac{Y-Z}{Y}\times 100\%$$

where *σ*_1_ and *σ*_2_ are the standard deviation of detected signal and de-noised signal, *Y* and *Z* are the maximum amplitude of detected noisy signal and de-noised signal. High NRR and low ARR means effective de-noising results. The evaluation parameters using each de-noising method are given in Table 6. The result shows that the proposed method is capable of suppressing noise effectively with less amplitude reduction.

The computation of proposed de-noising method was carried out by MATLAB codes in computer platform of oscilloscope. The calculating time of de-noising two UHF PD signals is 4.751 s and 4.897 s, respectively. It means that the proposed de-noising method is feasible in field test, and the de-noised results can be obtained in real time.

## 7. Conclusions

To suppress the periodic narrowband noise and Gaussian white noise in UHF PD signal, a novel de-noising method is proposed based on the generalized S-transform and MTFM in this paper. The results are concluded as follows:

(1) To suppress periodic narrowband noise, the SMMV method based on generalized S-transform and MTFM is employed. By calculating the frequencies and amplitudes of the narrowband noise, the corresponding MTFMs are obtained and removed.
(2) To suppress Gaussian white noise, singular values are calculated through decomposing the 2-dimensional MTFM based on the generalized S-transform. Effective singular values can be selected successfully by employing the FCM clustering method.
(3) De-noising results of simulated and field detected UHF PD signals validate the feasibility of this method. Compared with some conventional de-noising methods, the proposed method obtains de-noised signals with high SNR and less waveform distortion.

## Figures and Tables

**Table 1:** Parameters of the simulated UHF PD signals.

PD Pulse Sequence	1	2	3	4
***τ*/ns**	0.5	1	0.5	1
***f*_c_/MHz**	600	600	1000	1000
***A*/mV**	10	40	10	40

**Table 2:** Calculated amplitudes and frequencies with changing values of *λ*.

*λ*	Narrowband Noise #A		Narrowband Noise #B		Narrowband Noise #C	
	Frequency (MHz)	Amplitude (mV)	Frequency (MHz)	Amplitude (mV)	Frequency (MHz)	Amplitude (mV)
**0.1**	470.085	1.949	900.032	1.919	1800.194	1.842
**0.2**	470.085	1.954	900.011	1.925	1800.194	1.883
**0.3**	470.085	1.986	900.076	1.931	1800.894	1.912
**0.4**	470.312	1.984	899.391	1.933	1802.140	1.924
**0.5**	470.476	1.979	901.328	1.926	1802.954	1.927
**0.6**	470.851	1.974	898.398	1.915	1803.823	1.925
**0.7**	470.994	1.976	901.852	1.929	1804.974	1.916
**0.8**	471.265	1.968	902.347	1.924	1805.293	1.917
**0.9**	471.325	1.968	903.481	1.919	1806.905	1.913
**1.0**	471.785	1.962	903.911	1.918	1807.488	1.904
**1.1**	472.046	1.953	904.056	1.911	1809.226	1.901

**Table 3:** Conventional de-noising methods.

Method	Noise Type	Technique
A	Narrowband noise + White noise	Proposed method in this paper
B	Narrowband noise + White noise	Adaptive IIR filter
C	Narrowband noise + White noise	Adaptive threshold WT de-noising (db2)
D	Narrowband noise + White noise	Adaptive threshold WT de-noising (db8)
E	White noise	Conventional SVD de-noising method

**Table 4:** De-noising evaluation parameters using each method.

Evaluation Parameter	De-Noising Method	Pulse 1	Pulse 2	Pulse 3	Pulse 4	All
SNR	Method A	14.445	19.482	16.794	20.987	16.473
	Method B	2.875	1.832	3.892	2.968	2.712
	Method C	−0.871	−0.362	−1.796	−1.704	−1.647
	Method D	0.585	0.315	1.607	1.861	0.941
	Method E	6.2548	7.629	8.662	9.029	8.342
RMSE	Method A	0.0013	8.3 × 10^−4^	7.6 × 10^−4^	3.7 × 10^−4^	3.4 × 10^−4^
	Method B	0.0248	0.0418	0.0196	0.0327	0.0198
	Method C	0.0587	0.0697	0.0234	0.0212	0.0432
	Method D	0.0551	0.0497	0.0189	0.0212	0.0347
	Method E	0.0124	0.0133	0.0166	0.0390	0.0134
NCC	Method A	0.9898	0.9942	0.9934	0.9970	0.9907
	Method B	0.7416	0.6446	0.8118	0.7256	0.5773
	Method C	0.3503	0.4506	0.5122	0.5418	0.4301
	Method D	0.4152	0.5738	0.6577	0.7479	0.6209
	Method E	0.8832	0.9112	0.8035	0.5389	0.8251
VTP	Method A	1.0745	1.0382	1.0260	1.0515	1.0476
	Method B	0.7814	0.6767	0.7042	0.7237	0.7432
	Method C	1.7645	1.2887	1.8554	1.7592	1.6346
	Method D	1.1484	1.0606	1.4327	1.3084	1.2636
	Method E	1.0852	1.0790	0.7157	0.7880	0.8956

**Table 5:** Calculated frequencies and amplitude of narrowband noise in field detection.

Signal	Frequency (MHz)	Amplitude (mV)
UHF signal 1#	875.9	1.03
	941.1	2.16
UHF signal 2#	876.4	3.53
	961.3	1.24

**Table 6:** De-noising evaluation parameters of field detected signals.

Signal	De-Noising Method	Evaluation Parameter	
		NRR	ARR
PD Signal #1	Method A	15.96	21.4%
	Method B	10.59	34.6%
	Method C	13.21	27.6%
	Method D	13.36	41.3%
PD Signal #2	Method A	19.23	32.5%
	Method B	17.25	48.3%
	Method C	16.68	43.4%
	Method D	17.66	36.6%

## Abbreviations

The following abbreviations are used in this manuscript:

PD	Partial discharge
UHF	Ultrahigh frequency
HV	High voltage
WT	Wavelet transform
SNR	Signal noise ratio
MTFM	Module time-frequency matrix
FCM	Fuzzy c-means
SMMV	Sub-matrix maximum module value method
SVD	Singular value decomposition
PRPD	Phase resolved partial discharge
CPU	Central processing unit
RAM	Random access memory
RMSE	Root mean square error
NCC	Normalized correlation coefficient
VTP	Variation trend parameter
RVTP	Rise variation trend parameter
FVTP	Fall variation trend parameter
NRR	Noise reduction ratio
ARR	Amplitude reduction ratio
